# Simulation for training in resource-restricted countries: using a scalable temporal bone surgical simulator

**DOI:** 10.5116/ijme.57c1.4c8a

**Published:** 2016-09-08

**Authors:** Gregory J. Wiet, Don Stredney, Thomas Kerwin, Bradley Hittle, D. Richard Kang

**Affiliations:** 1 Department of Otolaryngology, Nationwide Childrens Hospital and The Ohio State University, Columbus, Ohio, USA; 2 Ohio Supercomputer Center, Columbus, Ohio, USA; 3 Department of Otolaryngology, Boystown National Research Hospital, Omaha, Nebraska, USA

**Keywords:** Simulation, training, resource-restricted countries, temporal bone surgical simulator

## Introduction

Many resource-restricted countries suffer from poor access to healthcare. Often, even when the healthcare system is accessible, there is a lack of specialized expertise (in particular surgical), severely limiting treatment options. Access to advanced training in specific areas is either limited, or nonexistent. This is particularly evident in specialized surgical fields that employ laparoscopic and microsurgical techniques.  These techniques require not only cognitive knowledge, but also resources to obtain psychomotor training. The lack of specialized facilities negatively impacts deliberate training needed to obtain proficiency, and ultimately adversely effects specialized surgical care delivery.

To overcome the training shortcoming, local physicians may choose to train in other countries where more advanced training is available. Barriers to training abroad include cost, political will, and issues related to visas and certifications that regulate hands-on experiences. These limiting factors make this option available to only a small fraction of practitioners. Additionally, physical training facilities such as cadaveric or animal laboratories may not be available. The resultant deficiency in training perpetuates the lack of skill present and results in a nearly insurmountable problem in providing specialized surgical training to indigenous physicians. One of the keys to generating a significant impact in countries lacking specialized surgical expertise is with active training programs within the country’s existing training structure. We are exploring cost-effective surgical simulation to provide the necessary means for deliberate practice for specialized surgical training in resource-restricted countries.

All surgical expertise requires extensive deliberate practice with feeback.^1^ This concept is no more clearly demonstrated than in the development of skill in temporal bone (middle ear and mastoid) surgery. Temporal bone surgery requires microsurgical techniques in small confines with vital structures at risk such as hearing and balance end organs, the carotid artery and jugular vein and central nervous system structures in the middle and posterior fossa. Cognitive training can be obtained from textbooks and often time physicians in developing countries have adequate knowledge. The development of the psychomotor skills required of these procedures is much more difficult for these individuals to achieve. Traditionally, psychomotor skill in otologic surgery has been obtained within the cadaveric temporal bone laboratory. These facilities however, are extremely cost prohibitive (costing up to $ 1 million US) and, if such a facility were available, maintenance and preservation of equipment can prove unobtainable. Secondly, the acquisition and use of human temporal bone specimens presents a second and perhaps more insurmountable obstacle to this specialized surgical training. Facilities and resources to properly organize, harvest, preserve and distribute human tissue simply do not exist in resource-restricted countries.

With the advent of simulation technology, the ability to obtain the psychomotor training may be realized. We present a low-cost simulation environment that may help mitigate the impediments to deliberate rehearsal required for surgical expertise in otologic surgery. The purpose of this manuscript is to highlight our experience with an informal temporal bone course utilizing a portable/cost effective computer based temporal bone simulator in Managua, Nicaragua. By doing so, we help to inspire others in resource rich countries to bring to bare their expertise in their respective areas and to use cost effective methods to simulate both cognitive and psychomotor skills for training physicians in developing countries.

### Experience gained

Our pediatric Otolaryngology Division has been providing humanitarian aid to the people of Nicaragua. One of the primary conditions requiring our expertise has been in the area of otologic surgery. Local physicians in practice and in residency have very limited access to otologic training and therefore have limited capacity to care for patients requiring advanced ear procedures. They possess excellent cognitive knowledge; yet lack the psychomotor training necessary to provide adequate care for their patients. As a result, patients often suffer from complications related to otologic disease therefore making their treatment even more difficult and leading to greater morbidity, and at times, mortality related to otologic conditions.

The virtual temporal bone system developed at The Ohio State University and Nationwide Children’s Hospital provides a virtual environment similar to that provided in a cadaveric temporal bone laboratory.^2^^-^^6^ The representation of the temporal bone is based on CT image data; 40 different bones are available for training, 20 left and 20 right. The interface consists of a computer monitor and haptic (sense of touch) feedback device. The bone is displayed on the computer monitor and the haptic device provides a “three dimensional mouse” to allow menu selections, i.e., rotate the bone in any plane as well as emulation of the drill. The sound of the drill is modulated providing spatial cues through aural feedback. Using the haptic device, the user selects a bone, burr type (cutting vs. diamond), burr size, magnification and orientation of the bone. Depressing a button on the haptic device activates burr rotation.  The user may drill anywhere on the bone. Additional instructional elements are provided which include the capability to highlight specific structures, tool tips for viewing landmark labels, arbitrary sectioning of the data, and transparency of bone adjustment to view deep structures that have not been dissected. For transportability, the system was scaled down to perform in real-time on a laptop computer. A physical interface to emulate the drill provides six degree's of movement and three degree's of haptic feedback.  The total system costs approximately $5000.

Recently, our group returned to not only provide care for the population but also to trial the scaled down version of the virtual temporal bone simulator for use in an informal temporal bone dissection course. A makeshift “temporal bone laboratory” was set up in the OR nurse manager’s office at Lenin Fonseca Hospital in Managua, Nicaragua. Residents took turns using the virtual temporal bone dissection device to dissect virtual temporal bones using the steps outlined in Nelson’s Dissector.^7^ Faculty provided formative feedback during the practice sessions. Immediately after training, residents then scrubbed into surgery as first assistants in a mastoidectomy procedure.

Since this was an informal session and IRB approval was not requested, data acquisition as to the utility of the system in learning temporal bone surgery was not performed. Anecdotally, residents were thrilled with the experience, as none had ever executed these techniques either in a cadaveric lab or on a live patient.  It was our observation compared to previous trips that the residents were far more engaged in the live operation and were able to assist in a more efficient manner.

## Conclusions

The use of simulation is clearly on the forefront of surgical training in the United States and other developed countries. This applies to both low technology “box trainer” types to more sophisticated computer based systems. It may however, provide a more powerful and far reaching impact on resource-restricted countries. The challenge is to develop curriculums, methods and systems that are of low enough cost and of sufficient convenience to function in the developing world.

The use of a relatively low cost and portable computer based surgical simulator for teaching temporal bone surgery has been demonstrated. This methodology may provide a significant impact for surgeons in developing/resource-restricted countries with the means to advance their surgical skills and subsequently impact patient care in their countries. Development and use of simulation technologies and methodologies that are low cost, portable and demonstrate validity in training are needed. The impact may be significant in resource-rich countries but may provide even further impact in resource-restricted countries. The authors encourage others to develop low cost simulation based training programs that can be easily implemented in resource-restricted areas where access to training may be limited. The use of low cost simulation, particularly for psychomotor skills, may have significant impact in medical education in these countries.

### Conflicts of Interest

The authors declare that they have no conflict of interest.
